# Death Due to Caffeine and Methamphetamine Toxicity: A Case Report

**DOI:** 10.7759/cureus.66634

**Published:** 2024-08-11

**Authors:** Dae Geon Kim, Stephanie Powers, Lorenzo Gitto

**Affiliations:** 1 Medicine, Pusan National University School of Medicine, Yangsan, KOR; 2 Pathology, Cook County Medical Examiner’s Office, Chicago, USA

**Keywords:** autopsy, caffeine, methamphetamine, toxicology, acute toxicity

## Abstract

Caffeine (1,3,7-trimethylxanthine) is an over-the-counter psychostimulant that, when used in appropriate amounts, is generally considered safe. However, excessive use can cause various symptoms and, in severe cases, can even be life-threatening. A 34-year-old man with a reported history of psychiatric disorders was found unresponsive at his girlfriend’s house and transported to an emergency department. He was presumed to have taken several caffeine pills and was pronounced dead approximately six hours later. There was no evidence of trauma or natural diseases at autopsy. Toxicology testing on hospital blood samples revealed toxic levels of caffeine and methamphetamine. After investigation of the circumstances surrounding the death and accounting for the autopsy and toxicology findings, the cause and manner of death were certified as combined caffeine and methamphetamine toxicity and accident, respectively. Lethal levels of caffeine have been reported when blood concentration exceeds 80 mg/L. Caution is needed to avoid excessive caffeine intake, especially when consumed in concentrated forms like tablets or powders. Caffeine should be used with care not only in cases of cardiovascular disease or genetic vulnerability but also for those with psychiatric disorders. Although deaths from caffeine are rare, they are consistently reported, necessitating attention and caution in its use.

## Introduction

Caffeine (1,3,7-trimethylxanthine) is a well-known psychostimulant that is generally considered safe and effective, readily accessible in various natural sources such as coffee beans, tea leaves, and cacao pods, and commonly added to beverages and medications. Its stimulating effects on the central nervous system and cardiovascular system make it a popular choice for boosting alertness and combating fatigue.

Research indicates that 85% of Americans consume caffeine daily, averaging an intake of 165 mg per day [1]. Moderate consumption can enhance arousal, alertness, and concentration. However, excessive use can lead to cardiovascular (e.g., hypertension, arrhythmias, myocardial infarction) and psychological (e.g., anxiety, irritability) symptoms, and in severe cases, intoxication or even death [2-3]. Symptoms of caffeine intoxication range from anxiety and insomnia to life-threatening conditions. Detecting high levels of caffeine in the blood is crucial for accurate diagnosis in cases of toxicity, as distinct diagnostic symptoms may not be present.

The case presented involves a fatality attributed to toxic levels of caffeine found in the deceased's blood.

## Case presentation

A 34-year-old White male, with a medical history including bipolar disorder, schizophrenia, and fetal alcohol syndrome, was discovered unresponsive by his girlfriend on the living room floor of his residence. According to her, he had ingested approximately eight 200 mg caffeine pills and those pills were also found at the scene. He occasionally smoked marijuana and consumed one pack of cigarettes daily. Upon admission to the hospital, he tested positive for amphetamines. Despite efforts in the medical intensive care unit (MICU), he was pronounced dead approximately six hours later.

A forensic autopsy was conducted, revealing a body weight of 155 pounds and a height of 72 inches, resulting in a body mass index (BMI) of 21 kg/m². No recent significant injuries were evident. The heart weighed 442 grams, and the coronary arteries showed no significant atherosclerosis. Evidence of a patent foramen ovale was noted. The gastric lumen contained approximately 150 mL of grainy brown fluid. The left kidney exhibited a finely granular appearance, while other organs appeared unremarkable. Toxicology reports from hospital blood samples indicated caffeine levels of 180 mg/L, methamphetamine levels of 0.099 mg/L, and lorazepam levels of 0.023 mg/L (Table 1).

**Table 1:** Toxicology findings ^a^: reference ranges from Handbook of Forensic Toxicology for Medical Examiners [4]

Analysis	Results	Units	Source	Therapeutic/nontoxic range^a^	Lethal range^a^
Caffeine	180	mg/L	Blood	0.2-11 mg/L	13-50 mg/L
Methamphetamine	0.099	mg/L	Blood	0.02-7.5 mg/L	0.1-69 mg/L
Lorazepam	0.023	mg/L	Blood	0.01-0.2 mg/L	-

Based on his medical history, the circumstances surrounding his death, autopsy findings, and toxicology results, the cause of death was determined to be due to combined caffeine and methamphetamine toxicity, with the manner of death certified as an accident.

## Discussion

Deaths from caffeine toxicity, although rare, are consistently documented in the literature [5-8]. When orally ingested, caffeine is absorbed and metabolized into paraxanthine, theobromine, and theophylline. It acts primarily as a non-selective antagonist on adenosine A1 and A2A receptor subtypes, leading to various physiological effects including cardiovascular and psychological symptoms [2]. Symptoms such as anxiety, irritability, palpitations, arrhythmia, and seizures can manifest when blood concentrations exceed 15 mg/L, with fatal cases reported at concentrations exceeding 80 mg/L [9].

Dangerous blood levels are more commonly associated with the overuse of caffeine pills or tablets [3]; fatalities often result from consuming caffeine in concentrated forms like these [6], highlighting the need for caution with these products. In the case presented, circumstantial evidence suggests ingestion of caffeine in tablet form; while no intact or fragmented tablets or pills were found in the stomach, the gastric contents appeared grainy, potentially indicating residual tablet or pill sediment. Orally ingested caffeine pills are rapidly absorbed, typically within an hour, which could explain the observed appearance of the gastric contents.

Previous research [10] has indicated that by knowing the weight of the subject and the postmortem concentration of caffeine, it is possible to estimate approximately the amount of substance in the body at the time of death. Given the known volume of distribution of caffeine as 0.7 L/kg, a deceased weight of 155 pounds (approximately 70 kg), and a blood caffeine concentration of 180 mcg/mL, using the formula (0.180 g/L × 0.7 L/kg × 70 kg) it could be estimated that approximately 8.8 g of caffeine was absorbed and distributed in the body. Since the tablets found at the scene contained 200 mg each, it would take approximately 40 tablets to account for a caffeine blood concentration of 180 mg/L; other forms of caffeine intake could not be ruled out. Such calculation is generally considered controversial in postmortem samples because it does not consider the effect of postmortem redistribution of caffeine. In this case, toxicology analyses were performed on blood specimens collected from the subject while still alive, albeit in critical condition, virtually eliminating the postmortem redistribution process, making the estimation more reliable.

According to the literature, individuals with preexisting cardiovascular diseases are known to be at increased risk of experiencing symptoms at lower doses of caffeine [2-3,5]. In the case described, the deceased had a heart weighing 442 grams, which exceeded the 90th percentile for his age, sex, height, and weight, indicative of cardiomegaly. Cardiomegaly can arise from various conditions such as hypertension, valvular heart disease, cardiomyopathy, chronic substance use, and other heart abnormalities. Enlargement of the heart can affect its electrical signaling pathways, heightening the risk of cardiac arrhythmias.

Caffeine consumption typically results in a mild elevation of both systolic and diastolic blood pressure, affects heart rate (leading to either bradycardia or tachycardia depending on dosage), and stimulates neuroendocrine responses including the release of epinephrine, norepinephrine, and renin. In individuals with underlying heart conditions, particularly when consuming high doses, the stimulant effects of caffeine may exacerbate the risk of cardiac arrhythmias, potentially leading to fatal outcomes.

Another interesting aspect of this case is the deceased's history of multiple psychiatric disorders. While direct causation has not been established, evidence suggests that psychiatric conditions may indirectly heighten the risk of life-threatening events [11]. Given that caffeine can exacerbate psychiatric symptoms and interact unfavorably with psychiatric medications, it is crucial to consider the potential toxicity of caffeine not only in individuals with physical illnesses but also in those with psychiatric disorders. In psychiatric patients, caffeine toxicity can be compounded when concurrent health issues like cardiovascular disease are present.

Caffeine metabolism primarily involves cytochrome P450 1A2 (CYP1A2), a liver enzyme crucial for its breakdown. Genetic variations in the CYP1A2 gene can influence individual caffeine metabolism rates. Those with genetically slower caffeine metabolism may experience prolonged effects and heightened sensitivity to caffeine, potentially increasing the risk of conditions like myocardial infarction [12]. Although there was no reported history of CYP1A2 gene polymorphism in this case, genetic testing could be considered if a blood sample is collected in a purple-top tube (ethylenediaminetetraacetic acid (EDTA)) during autopsy.

In this case, the caffeine concentration in the blood was 180 mcg/mL, which is significantly above the reported lethal dose. Methamphetamine was also detected, but its concentration alone was not consistent with an isolated acute toxicity. Nevertheless, since methamphetamine is also a psychostimulant, a synergic effect with caffeine should be considered in the determination of the cause of death [13]. Both substances are stimulants that affect the central nervous system, and when used together, they can amplify each other's effects, leading to heightened stimulation and potential cardiovascular and psychiatric complications. The combination of methamphetamine and caffeine can significantly increase heart rate, blood pressure, and cardiac workload. This heightened cardiovascular stress can precipitate serious events such as myocardial infarction, arrhythmias, hypertensive crisis, and stroke, potentially leading to sudden cardiac death.

## Conclusions

This case report discusses a death attributed to caffeine and methamphetamine toxicity and explores aspects of caffeine intoxication. While current literature generally considers caffeine intake from typical food and beverage sources safe for healthy individuals, certain groups, such as those with preexisting cardiovascular disease or psychiatric disorders, are at higher risk of experiencing harmful effects from caffeine.

Although deaths from caffeine toxicity are rare, they are periodically reported, underscoring the importance of ongoing caution and awareness. In forensic pathology, toxicology reports may indicate caffeine levels as positive or negative. When no toxic levels of other substances are found during toxicology analysis, forensic pathologists should consider the possibility of elevated caffeine concentrations. High caffeine levels can provide crucial information in cases where no apparent cause of death is initially identified, following a comprehensive medicolegal death investigation.
